# A case report of racemose pattern of intracranial tuberculoma with brain stem and hypophyseal involvement developing paradoxically during treatment.

**DOI:** 10.1259/bjrcr.20160034

**Published:** 2016-07-28

**Authors:** Denver Steven Pinto, Thara Joseph, Arun George, Ravi V Hoisala

**Affiliations:** Department of Radiodiagnosis, St John’s Medical College, Bangalore, India

## Abstract

Tuberculosis involving the central nervous system, a source of considerable morbidity and mortality, forms 5-10% of the disease burden associated with tuberculosis. Central nervous system tuberculosis may present as meningitis, tuberculoma, abscesses, cerebritis or miliary tuberculosis. The most common site of tuberculoma has been reported to be at the grey–white matter junction and the periventricular region. They may even be found in the epidural, subdural and subarachnoid spaces, and the brain stem, with the rarer sites of involvement being the cavernous sinus, sella turcica, hypophysis, hypothalamus, sphenoid sinus and the mastoid air cells. Although tuberculosis is very common in developing countries, with the increasing prevalence of immunosuppression owing to human immunodeficiency virus and patients surviving chemotherapy or organ transplantation, the incidence of tubercular infections has been rising in developed countries. The authors report a case of intracranial tuberculosis in a human immunodeficiency virus-negative patient, who underwent incomplete treatment for tubercular peritonitis and presented with unilateral ptosis. Tuberculous involvement was noted in a racemose pattern in the subarachnoid space, cavernous sinuses, suprasellar cistern and parasellar region. To the best of our knowledge, the term racemose pattern of tuberculoma has not been described before, while about 10 cases of tuberculoma involving the cavernous sinuses have been reported in the literature. Furthermore, the racemose pattern of tuberculosis in the subarachnoid space, as well as involvement of the cavernous sinus, hypothalamus, pituitary and the cisterns, developed paradoxically after initiation of antitubercular chemotherapy.

## Summary

Tuberculosis is the seventh leading cause of morbidity and mortality worldwide and has reached epidemic proportions in both developed and developing nations. 5-10% of tuberculosis cases involve the central nervous system (CNS).^1^ Intracranial tuberculoma is the result of haematogenous spread from a primary lung focus, which may be quite small and therefore not identifiable on routine chest radiographs.^2^ Haematogenous spread of tuberculosis to the CNS may present as tuberculoma, meningitis, cerebritis, abscess or miliary tuberculosis.^3,4^

While tuberculomas may reduce in size or become invisible on imaging studies, in some cases, clinical deterioration with development of new or enlargement of existing lesions may occur despite recovery from meningitis. This worsening in neurotuberculosis has been attributed to a paradoxical response and may occur within days or even 1 year after starting chemotherapy, despite regular antitubercular chemotherapy^5^. However, it is difficult to differentiate a paradoxical reaction from drug resistance or relapse of the disease.

We present a case of tuberculoma involving the subarachnoid spaces in a racemose pattern, cavernous sinuses, midbrain, hypothalamus, pituitary, suprasellar cisterns and parasellar region, which developed with parenchymal tuberculomas and tuberculous meningitis (TBM) after beginning treatment with antitubercular chemotherapy.

## Case history, clinical findings and imaging

**Figure 1.**
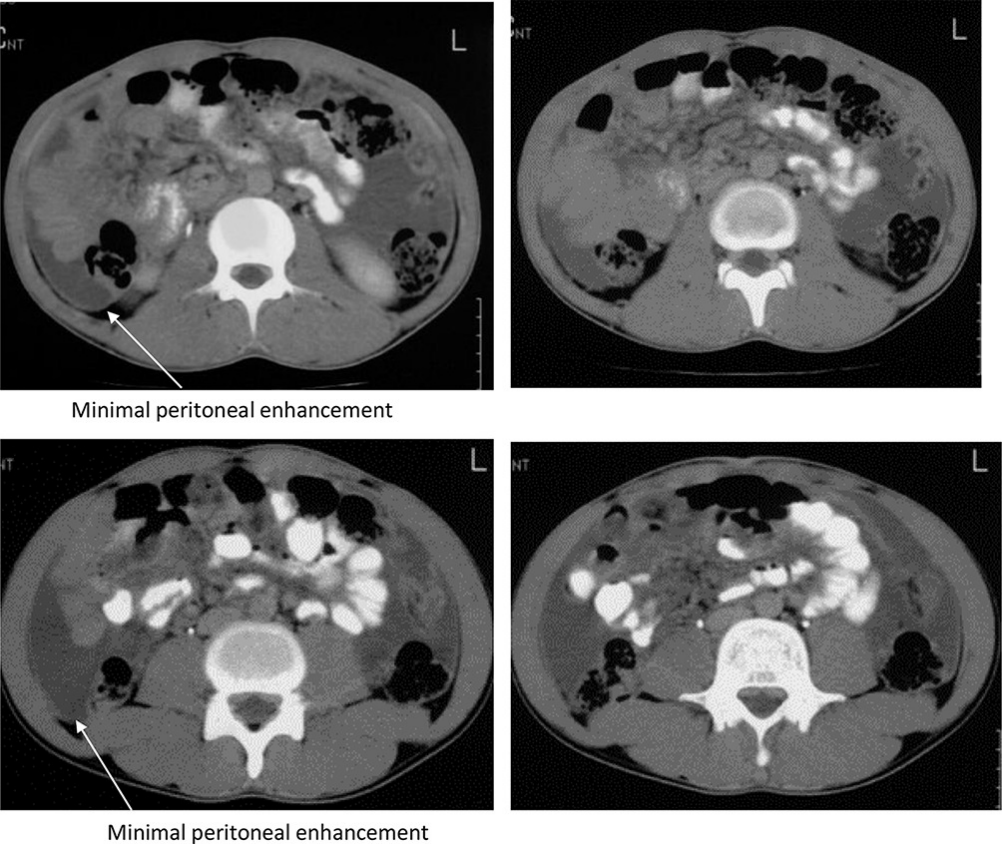
Axial CT images of a patient presenting with abdominal distension and pain showing ascites with minimal peritoneal thickening and enhancement. A diagnosis of tubercular peritonitis was made, following which an ascitic tap was performed. Culture of the ascitic fluid showed growth of *Mycobacterium tuberculosis*. Thus, the diagnosis of tubercular peritonitis was confirmed.

A 26-year-old male patient of Indian origin who had previously defaulted on his treatment for tuberculous peritonitis (Figure 1), without other known comorbidities, presented with ptosis and down and outward deviation of the left eye. Clinical examination was positive for meningeal signs and complete left oculomotor nerve palsy.

A lumbar puncture was performed, which yielded clear fluid with lymphocytic pleocytosis with very low cerebrospinal fluid (CSF) glucose level of 18 mg dl^–1^ (normal 45–80 mg dl^–1^). CSF adenosine deaminase level was 23.3 (normal 1–9 IU l^–1^). CSF polymerase chain reaction detected *M. tuberculosis* and *M. tuberculosis* complex consisting of *M. bovis, M. microti and M. africanum*. Serology was negative for human immunodeficiency virus infection. Cysticercosis immunoglobulin G level was found to be 0.9 ( >1.1 ng dl^–1^ is considered positive).

**Figure 2.**
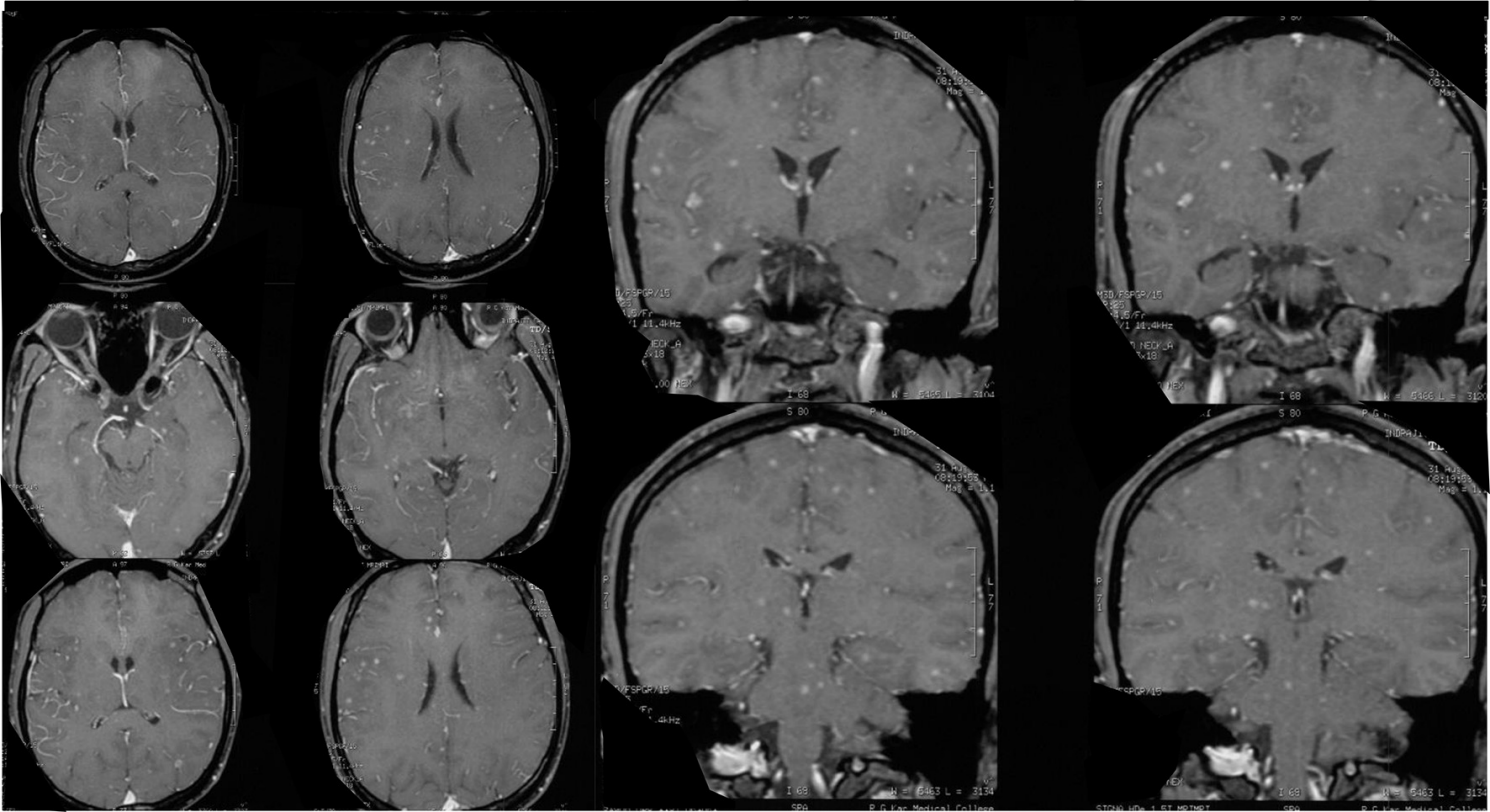
Post-contrast axial (left) and coronal *T*_1 _weighted images (rightb) showing multiple nodular enhancing lesions in bilateral temporal and parietal regions, preferentially involving the grey–white matter junction, the midbrain and pons of the brain stem, with tuberculous meningitis evidenced by leptomeningeal enhancement.

MRI of the brain with contrast performed at this time showed multiple parenchymal nodular enhancing lesions in all the lobes of the bilateral cerebral hemispheres with mild surrounding oedema. (Figure 2) A diagnosis of multiple parenchymal tuberculomas with TBM was made. The patient was restarted on antitubercular therapy (ATT) with the addition of steroids.

The left oculomotor nerve palsy clinically resolved with treatment. Repeat MRI performed 1 month later showed persisting basal meningeal enhancement with disappearance of most of the tuberculomas. However, the patient continued to have recurrent headaches. He completed the 9-month treatment course for the tuberculomas, but at follow-up 4 months after the conclusion of treatment, he presented with incontinence of urine for the past 2 months and one episode of generalized tonic–clonic seizures.

**Figure 3.**
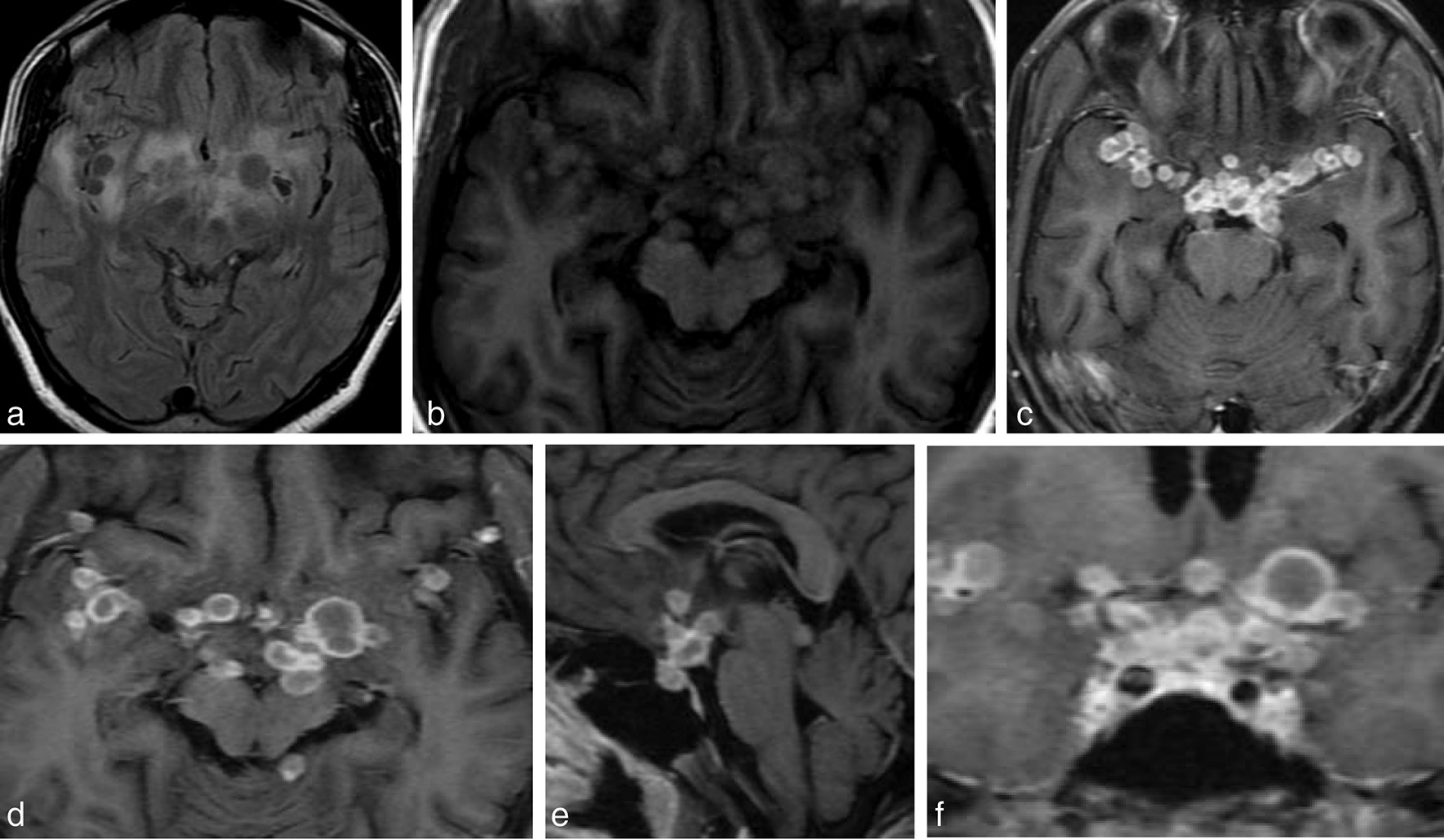
(a, b) *T*_2 _fluid-attenuated inversion-recovery and *T*_1_ weighted axial images show lesions that are *T*_2_ hypointense to the cortex, mildly *T*_1_ hyperintense to the cortex and isointense to the white matter at the grey–white matter of bilateral insular cortices with perilesional oedema. Lesions are also noted in the sylvian fissure and the prepontine cistern involving the left cerebral peduncle. (c, d) Axial *T*_1_ post-contrast images showing ring-enhancing lesions, with involvement of the sylvian fissure in a racemose pattern. (e) Sagittal *T*_1_ post-contrast image showing ring-enhancing lesions in the pituitary. (f) Coronal *T*_1_ post-contrast image showing involvment of the cavernous sinus on both sides and the floor of the third ventricle.

A repeat MRI showed evidence of multiple rounded lesions that were mildly *T*_1_ hyperintense compared with the cortex and isointense compared with the white matter, and *T*_2_ hypointense compared with the cortex. There was perilesional oedema and ring enhancement of the lesions in a racemose pattern in bilateral insular cortices and sylvian fissures, with ring-enhancing lesions in the cavernous sinus, the pituitary and suprasellar cisterns, floor of the third ventricle, perimesencephalic cistern abutting the cerebral peduncles on both sides and the quadrigeminal cistern. Meningeal enhancement was present in the parasellar regions (Figure 3). This led us to suspect the possibility of recurrence of the tuberculomas, and the patient was started on antitubercular chemotherapy. A differential diagnosis of paradoxical response was considered.

**Figure 4.**
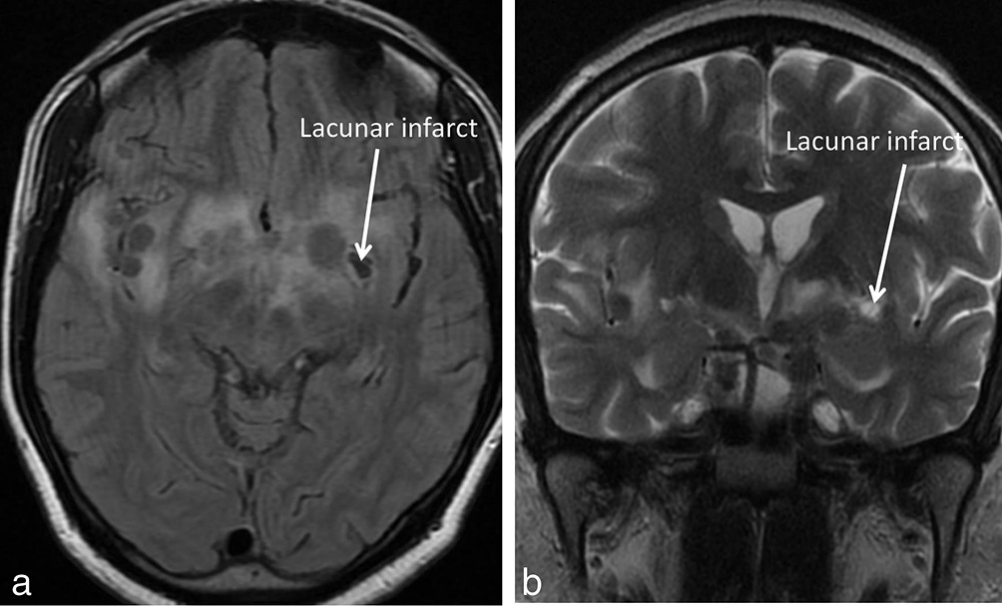
Axial *T*_2 _fluid-attenuated inversion-recovery and coronal *T*_2_ weighted images of the patient following an increase in the size of the tuberculomas (same time as Figure 3) shows a chronic lacunar infarct in the left gangliocapsular region.

At this time, a chronic lacunar infarct in the left gangliocapsular region was noted (Figure 4). Despite extensive history taking and examination, no symptoms were attributable to this lacunar infarct.

**Figure 5.**
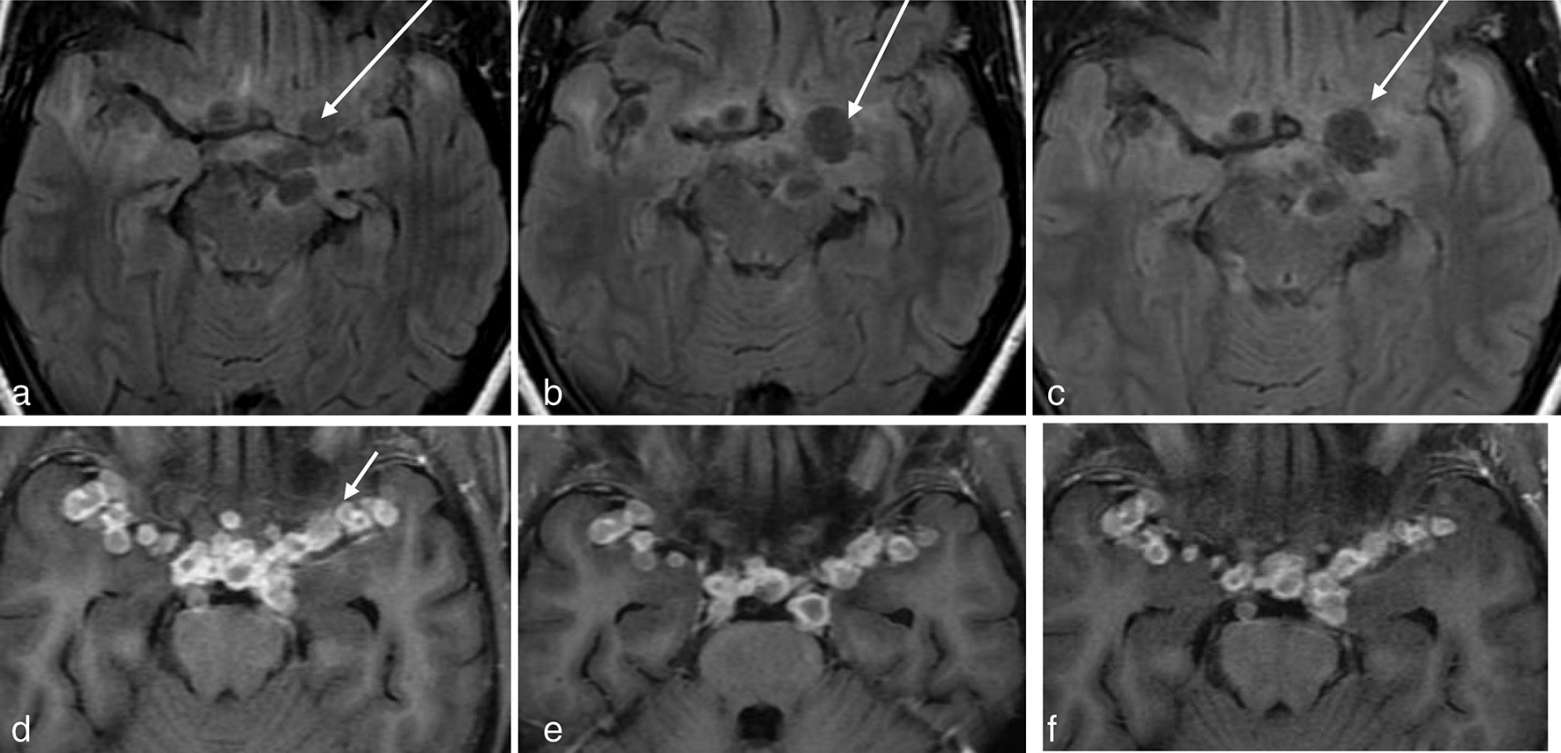
(a–c) Axial *T*_2 _fluid-attenuated inversion-recovery images at 6-month intervals showing mild interval change. The lesion marked with the long arrow is seen to show an interval increase in size despite treatment, while the lesion at the left cerebral peduncle is seen to maintain its size. (d–f) Images showing a racemose pattern (small arrow) of ring-enhancing lesions, which remain almost the same on serial imaging. Serial imaging has been performed thrice at 6-month intervals.

**Figure 6.**
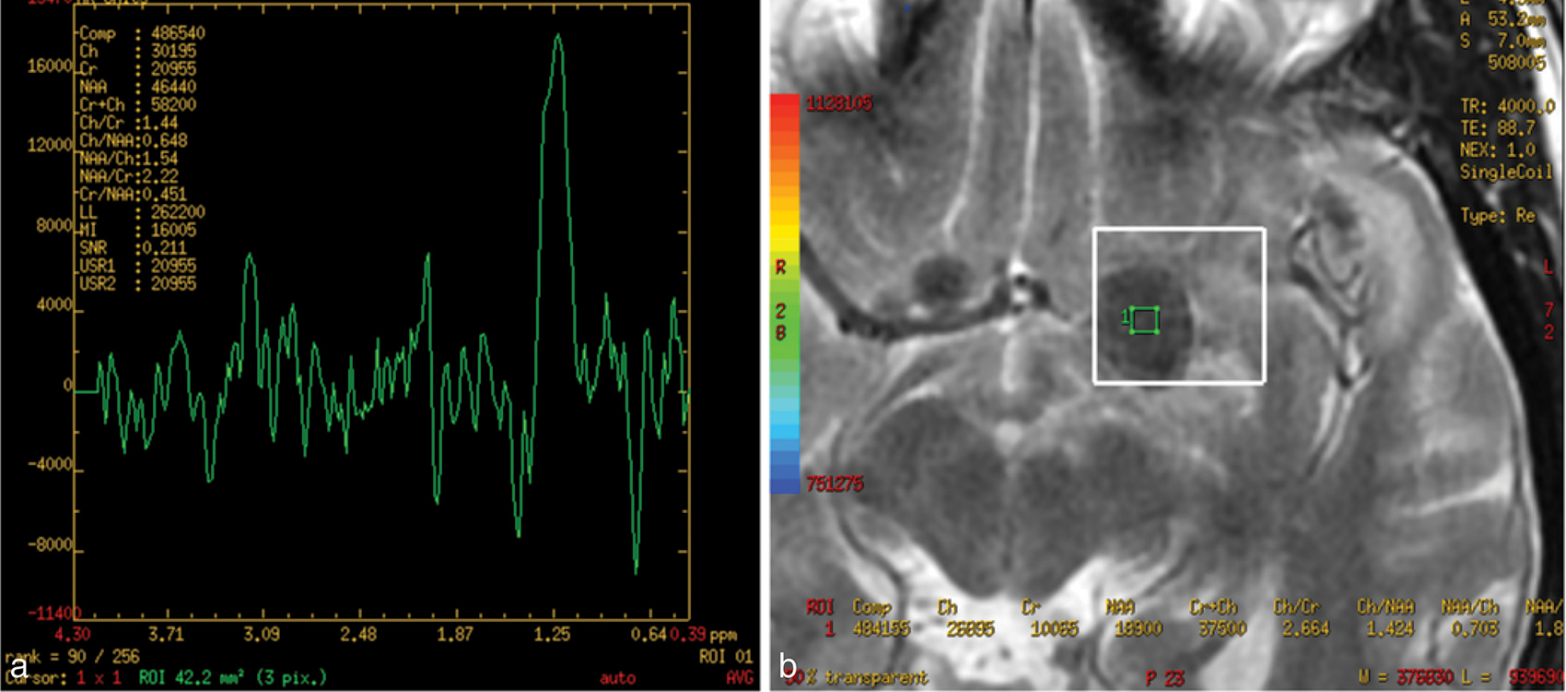
(a) MR spectroscopy image at the centre of a large tuberculoma showing elevated lactate peak at the third serial imaging. This confirmed central necrosis of the tuberculoma. (b) Image showing the placement of the region of interest.

A clinical decision to continue the antitubercular chemotherapy with corticosteroids was taken. Following this, the patient adhered strictly to the treatment regimen, which started with isoniazid, rifampicin, pyrazinamide, ethambutol and streptomycin for 6 months with corticosteroids, followed by maintenance therapy with isoniazid, rifamicin and ethambultol. Two further contrast-enhanced MRI studies of the brain were performed over the next year, 6 months apart (Figure 5). There was an increase in the size of one of the lesions, while the other lesions remained of similar size. There were no new lesions, no development of hydrocephalus or meningeal enhancement. MR spectroscopy (MRS) was performed owing to the lack of response to ATT. MRS showed evidence of reduced choline and *N*-acetyl-aspartate, with a lipid/lactate peak (Figure 6). The patient is currently asymptomatic on maintenance treatment with isoniazid, rifampicin and ethambutol with corticosteroids, with the ring-enhancing lesions showing minimal change over 12 months (three 6-monthly MRIs). A current lumbar puncture shows normal CSF cytology and biochemistry with normal adenosine deaminase levels (1.75 IU l^–1^).

## Discussion

Increase in the size or development of new brain lesions during or after the treatment of TBM, although uncommon, has been reported in the literature and is called a paradoxical response.^6–8^ It has been attributed to a Type IV hypersensitivity reaction developing within the tuberculoma, resulting in infarction, vasculitis and oedema.^9^ It is difficult to differentiate a paradoxical reaction from drug resistance or relapse of the disease. However, to call the increase in size or the number of tuberculomas a paradoxical response, there must be an initial response to treatment.^10^ In the case reported, there was clinical resolution of the left-sided oculomotor nerve palsy, with resolution of most of the tuberculomas, but the patient continued to have headache. However, with the earlier history of default on treatment of tubercular peritonitis, we had to consider a differential diagnosis of resistance.

The paradoxical response of CNS tuberculosis is a diagnosis of exclusion. For its definitive diagnosis, drug resistance needs to be excluded.^11^ The patient was treated adequately with first-line chemotherapeutic drugs for appropriate duration. After the initial diagnosis, there was no acid-fast bacilli in the CSF or growth of the tuberculous bacilli on CSF culture. Thus, we were compelled not to make a diagnosis of resistance to treatment.

Furthemore, in our case, the patient’s clinical symptoms resolved with no recurrence of symptoms but only persistence of the radiological abnormality. In a study of paradoxical CNS tuberculosis by Pepper et al,^12^ 6 (37%) of 16 patients had residual neurological deficit at the 6-month follow-up. Furthermore, this paper describes symptomatic resolution in patients at 6 months but the resolution of radiological features was not commented upon.

A case series of 10 patients by Gupta et al^13^ showed that, in one case, there was radiological deterioration in an asymptomatic patient. Furthermore, a case report by Sivadasan et al^14^ described rapid radiological evolution of an optic nerve tuberculoma with subtle paradoxical radiological worsening after initiation of ATT. This was followed by persistence of granuloma on a follow-up scan at 18 months.^14^ This case report by Sivadasan et al^14^ showed a tuberculoma that behaved similarly to the tuberculomas in the currently reported case, in its persistence on follow-up imaging. Furthermore, as there was no clinical deterioration of the patient in our case, the possibility of secondary resistance to tuberculosis was less likely. This is supported in the literature by a case report by Zaki et al.^15^

Furthermore, a lacunar infarct was noted in the left gangliocapsular region at the time of follow-up imaging following completion of 9 months of therapy (Figures 3 and 4). This finding has been supported in papers by Daset al^16^ and Koh et al.^17^ In a study on cerebral infarction in tubercular meningitis, Koh et al^17^ showed that 5 out of 38 patients had lacunar infarcts. This is probably owing to the vasculitis associated with tubercular meningitis.

Adjunctive corticosteroid treatment provides seizure control and reduces the tuberculoma size and number. Surgical therapy is provided when medical therapy fails, when surgical decompression is necessary or when the diagnosis is uncertain. The reason for a paradoxical reaction in an immunocompetent person is not known, but is supposed to have an immunological basis. The pathogenesis has been attributed to factors such as persistence of lipid-rich insoluble cell wall antigen infected tissue, exposure and release of new antigen targets during mycobacterial killing, hypersensitivity and exaggerated immune restoration occurring during the treatment of tuberculosis.^16^

Our case report is unique in its presentation of multiple tuberculomas in the subarachnoid space in a racemose pattern, with involvement of the cavernous sinus and the pituitary, which are both rare sites for development of a tuberculoma, while the earlier reported cases have been of parenchymal, optochiasmatic and dural tuberculomas.^1,5,18^ Furthermore, the cavernous sinus and the pituitary are rare sites for tuberculomas, with less than 10 reported cases of cavernous sinus involvement.^19,20^ To the best of our knowledge, the term “racemose pattern of tuberculoma” has not been used before. The pattern of tuberculoma reported as “a racemose pattern” in this report has been seen in a few cases in the literature in basal TBM. However, based on its appearance on images, which was similar to the racemose pattern of neurocysticercosis, the authors refer to this imaging pattern as a racemose pattern of tuberculoma. MRS portrayed a lipid/lactate peak suggestive of a tuberculoma. Awareness of the phenomenon of appearance or enlargement of tuberculoma during successful treatment of TBM can avoid unnecessary panic, changes in drugs and surgery. Furthermore, categorization of enlargement or increase in the number of tuberculomas as resistance to treatment when there is no initial response to therapy will enable putting the patient on second-line therapy. However, it may not always be possible to definitely differentiate a paradoxical response from recurrence or even resistance. In this patient, although there was an increase in the size of the tuberculoma after initial resolution, on persistence of chemotherapy with corticosteroids, there was only minimal change in the size with relatively constant number of the tuberculomas.

## Conclusions

A paradoxical response during the treatment of intracranial tuberculosis is a diagnostic challenge for clinicians and radiologists. Clinicians should be aware of this entity so that antituberculous chemotherapy is continued under the cover of corticosteroids. However, it is difficult to differentiate a paradoxical response from resistance to treatment or recurrence. In the reported case, as there was only a minimal change in the size of the tuberculomas, a clinical decision to continue with antitubercular chemotherapy with corticosteroids was taken.

## Learning points

1. The important role of CSF polymerase chain reaction and MRS in the definite diagnosis of tuberculoma is stressed upon.
2. The diagnosis, follow-up and management of this interesting and uncommon phenomenon requires serial MRI of the brain and familiarity with the idiosyncrasies of tuberculosis infection.
3. Cavernous sinus and the pituitary are rare sites for the development of tuberculoma.
4. Racemose pattern is an important presentation of tuberculoma.

## Consent

Informed consent to publish this case (including images and data) was obtained and is held on record.
